# Age-friendly cities: challenges for future research

**DOI:** 10.2471/BLT.18.224865

**Published:** 2019-05-14

**Authors:** J Mark Noordzij, Mariëlle A Beenackers, Ana V Diez Roux, Frank J van Lenthe

**Affiliations:** a Department of Public Health, Erasmus University Medical Center, P.O. Box 2040 3000 CA Rotterdam, Netherlands.; b Department of Epidemiology and Biostatistics, Drexel University, Philadelphia, United States of America.

The development of age-friendly cities and communities has become an important area of work in the fields of public health, ageing and public policy. This development reflects several larger trends including the complexity of demographic change and the recognition of the role of the environment in healthy ageing.^1^

In 2017, there were an estimated 962 million people aged 60 years or older worldwide, that is, around 13% of the global population. This part of the population is growing at an annual rate of about 3% and further growth is almost inevitable.^1^ An increasing part of this population lives in cities, where the combination of urbanization and ageing leads to new public health challenges, such as a higher risk of mental disorders, resulting in impairments in the ability to function socially.^2^ However, while cities pose major challenges for older citizens, they also offer opportunities for the implementation of policies and interventions that promote public health. 

In 2006, the World Health Organization (WHO) initiated a programme specifically targeting the health of urban residents aged 60 years and older, linking the challenges of urbanization and ageing. This collaborative programme aimed to identify which features of the built and social urban environment are essential in creating sustainable and supportive environments for older residents, and culminated in the publication of the *Age-friendly city guide* in 2006.^3^

An age-friendly city was defined as a place that encourages active ageing by optimizing opportunities for health, participation and security to enhance quality of life as people age.^3^ Starting with 33 cities, WHO built on the guide by launching the Global Network for Age-friendly Cities and Communities in 2010, currently consisting of more than 500 cities where more than 155 million people live.

The network has reached several of the guide’s objectives, such as generating greater recognition of the implications of population ageing on urban planning and involving stakeholders at multiple governmental levels.^4^ At the same time, some of the network’s limitations must be considered, as age-friendly initiatives often compete with wider objectives associated with economic growth and development.^1^ Furthermore, exchange between the age-friendly city movement and related debates in urban geography, sociology and other social sciences remains limited. This gap is most notable around research on structural urban changes, such as the rise of global cities, widening socioeconomic inequalities, and the impact of rural migration.

With increasing population ageing and urbanization, the development of age-friendly environments is a topic that demands the attention of both researchers and policy-makers. Two approaches hold the potential to move age-friendly city research forward: integration of determinants of ageing at multiple levels and the dynamics of urban environments. 

## Determinants of ageing

The guide^3^ reflects on then-current scientific developments by including determinants of active ageing into its model, such as social determinants and the built environment. These determinants have become the focus of researchers looking at different factors that contribute to the age-friendliness of cities. A review of age-friendly city research concluded that most studies can be ranked on a set of axes ranging from physical to social environment on one axis, to bottom-up to top-down governance on the other.^5^ Most studies reviewed look either at outcomes that define the age-friendly city or at processes associated with age-friendly cities.

A recent publication builds on these findings by exploring the prevalence of the tendency “to focus on the characteristics of ageing individuals and their immediate milieu, while paying little attention to the interaction between the micro-individual traits and the macro-level workings.”^6^ However, we argue that the understanding of micro-individual traits is the beginning of age-friendly city research and not the endpoint. The layers of the sociospatial world are nested within one another: the household, the neighbourhood, the city and so on. The age-friendly city is uniquely positioned to improve our understanding of how these layers interact with one another and how the combined impact of these mechanisms influences ageing individuals. Assessing the combined effects of different micro and macro processes on ageing could provide valuable information on how to better adapt to the challenges of urban ageing.

An example of how micro and macro processes are intertwined in the age-friendly city context is that of the prioritization of working-age families in urban renewal processes. This prioritization marginalizes older people from urban renewal, which implicitly creates a cultural bias towards age-segregated residential landscapes.^7^ Micro levels such as the individual’s home environment cannot be explained adequately without considering such macro-level processes and the role they play in shaping the immediate home environment.

While many studies investigate micro-level indicators of age-friendly cities, such as accessibility or individual safety, relatively few studies investigate how large socioeconomic and sociospatial developments impact ageing communities and the cities they live in. An example is the seesaw effect between urban greenness and urban density: both an increase in the amount of urban green areas and the development of more compact cities have been linked to better health.^8^ What if increasing urban density leads to a reduction in the amount of urban green spaces? Will there still be a net benefit to public health? Considering how these concepts work together is important to determine how they influence health. Untangling such relational effects requires an integrated approach that considers both micro and macro levels, and therefore require the synthesizing of multiple topic areas and indicators from the guide to assess their combined effects.

As with many other public health challenges, a systems approach may be a promising strategy to analyse such effects. Two sample research questions exemplify this integration of cross-level interactions. First, what multilevel processes of sociospatial transformation are driving urban change and how do these forces impact the health of ageing urban populations? Second, how can we use system-based approaches to simulate the effect of prevention and early identification policies specific to urban environments on the trajectories of ageing and well-being? To answer these questions, engaging in current debates in the fields of urban geography and complex system science is necessary and can provide new perspectives.

## Dynamics of urban environments

A second important approach to consider relates to the temporal character of urban environments. Cities are dynamic and constantly evolving,^9^ but this dynamic character is often neglected in age-friendly city research, as most existing research commonly applies a static lens that assumes that the individual’s needs and capacities can be based on their current location. Temporal characteristics such as neighbourhood stability or the longevity of an individual’s residence in a specific place are essential dimensions to understand how environmental changes affect ageing individuals. Age-friendly city research would therefore benefit from more longitudinal or experimental study designs that account for this dynamic character.^10^

An example is the effect of life-course neighbourhood exposures on health. The life-course perspective informs understanding of how at later periods in life, health is affected by earlier experiences. This perspective also draws attention to historical circumstances and periods that are vital in shaping people and places.^11^ Tracking the individual’s personal geography through time will not only allow to improve measures of exposure, but also chart the socioeconomic trajectories of the places they inhabit. This trajectory is especially relevant for older individuals who by default have a more substantial life-course history, and the effects of their history may therefore be more pronounced than in younger individuals.

Two sample research questions are relevant to this approach. First, how are life-course histories of ageing individuals connected to the sociospatial histories of urban environments, and how does this combined effect influence health? Second, how do individual space-time constraints and temporal rhythms of activities affect health and health behaviour in ageing urban populations? Answering these questions will not only contribute to a better understanding of how the space-time of individuals and places relate to current health, but will also provide better insight on how age-friendly developments are influenced or limited by other historical urban developments. Building the evidence base this way will provide both researchers and policy-makers with tools to better design age-friendly communities.

The public health challenges of ageing and urbanization are likely to intensify in the coming decades. Age-friendly cities still hold potential for both researchers and policy-makers. This potential should be further explored as the age-friendly city will never be an achieved condition, but an offer of an open horizon to work towards sustainable and age-friendly environments.
